# Japanese encephalitis vaccine-facilitated dengue virus infection-enhancement antibody in adults

**DOI:** 10.1186/s12879-016-1873-8

**Published:** 2016-10-18

**Authors:** Yuka Saito, Meng Ling Moi, Nozomi Takeshita, Chang-Kweng Lim, Hajime Shiba, Kuniaki Hosono, Masayuki Saijo, Ichiro Kurane, Tomohiko Takasaki

**Affiliations:** 1 Department of Virology 1, National Institute of Infectious Diseases, Tokyo, 162-8640 Japan; 2 College of Bioresource Science, Nihon University, Fujisawa, Kanagawa 252-0880 Japan; 3 Department of Virology, Institute of Tropical Medicine, Nagasaki University, Sakamoto 1-12-4, Nagasaki, Nagasaki 852-8523 Japan; 4 National Center for Global Health and Medicine, Tokyo, 162-8655 Japan; 5 National Institute of Infectious Diseases, Tokyo, 162-8640 Japan; 6 Kanagawa Prefectural Institute of Public Health, Chigasaki, Kanagawa 253-0087 Japan

## Abstract

**Background:**

Dengue virus (DENV) and Japanese encephalitis virus (JEV) belong to the genus *Flavivirus*, and infection with a virus within this genus induces antibodies that are cross-reactive to other flaviviruses. Particularly in DENV infection, antibodies to DENV possess two competing activities: neutralizing activity and infection-enhancing activity. These antibody activities are considered central in modulating clinical outcomes of DENV infection. Here, we determined the neutralizing and infection-enhancing activity of DENV cross-reactive antibodies in adults before and after JE vaccination.

**Methods:**

Participants were 77 Japanese adults who had received a single dose of inactivated Vero cell-derived JE vaccine. A total of 154 serum samples were obtained either before or approximately a month after a single dose of JE vaccination. The antibody-dependent enhancement (ADE) activity to each of four DENV serotypes and the neutralizing activities to DENV and to JEV were determined in each of the serum samples by using baby hamster kidney (BHK) cells and FcγR-expressing BHK cells.

**Results:**

A total of 18 post-JE immunization samples demonstrated cross-reactivity to DENV in an anti-DENV IgG ELISA. DENV neutralizing antibodies were not detected after JE vaccination in this study. However, undiluted post-JE vaccination serum samples from 26 participants demonstrated monotypic and heterotypic ADE activity to DENV. ADE activity was also observed in 1:10-diluted samples from 35 of the JE vaccine recipients (35/77, 45 %).

**Conclusion:**

In summary, JE vaccination induced DENV cross-reactive antibodies, and at sub-neutralizing levels, these DENV cross-reactive antibodies possess DENV infection-enhancement activity. The results also indicate that cross-reactivity to DENV is associated with high levels of JEV neutralizing antibodies and, the DENV cross-reactivity is further facilitated by JE vaccination.

**Electronic supplementary material:**

The online version of this article (doi:10.1186/s12879-016-1873-8) contains supplementary material, which is available to authorized users.

## Background

Dengue virus (DENV) infection mainly occurs in tropical and subtropical areas. It is estimated that there are 390 million dengue infections annually, and recently the number of dengue cases has increased exponentially worldwide [1]. Although DENV had not previously been endemic in Japan, a dengue outbreak occurred in 2014, resulting in 162 autochthonous dengue cases [2]. DENV and Japanese encephalitis virus (JEV) co-circulate in many Asian countries [3]. JE occurs in East, South, and Southeast Asia. Both DENV and JEV cause serious public health threats and are the leading causes of hospitalization and death in children in vast regions of Asia. These viruses belong to the genus *Flavivirus*, and infection with a virus within this genus induces antibodies that are cross-reactive to other flaviviruses [4–6]. Particularly in DENV infection, antibodies induced has been demonstrated to possess 2 competing activities: neutralizing and infection-enhancement activities. Antibodies with DENV infection-enhancement activities are speculated to contribute to disease severity by a mechanism known as antibody-dependent enhancement (ADE). This mechanism leads to high levels of infection and the production of progeny viruses in DENV target cells, the FcγR-expressing monocytes and macrophages. Infection-enhancement activity also hampers virus neutralizing activity [7, 8].

DENV and JEV are antigenically related viruses. Cross-reactivity was observed in vitro between both of these viruses [4, 5]. An improved understanding of pre-existing immunity to JEV and the implications of this flavivirus-primed immunity in subsequent infection are important as DENV outbreaks often occur in JEV co-endemic areas as well as in areas where JE immunization is routine. Vaccination also induces cross-protection between JEV and viruses from the same Japanese encephalitis serocomplex-group including West Nile virus (WNV), and Murray Valley encephalitis virus in animal models [9, 10]. Natural DENV and JEV infection or JE vaccine immunization, could each contribute to the overall immune background of an individual in areas where both DENV and JEV co-circulate [11, 12]. Therefore, it is particularly challenging to define the association between pre-existing immunity to JEV and dengue clinical severity in DENV endemic areas. Although cross-protection against DENV was observed in mice inoculated with JE vaccine, previous investigators have also demonstrated that flavivirus antisera enhance DENV-2 infection [13]. These antibodies with neutralizing activity (NA) against JEV induced by either a prior JEV infection or by JE vaccination also possess neutralizing activity to DENV in vitro [5, 14]*.* However, other investigators reported absence of cross-protective neutralizing antibodies of viruses from the same serocomplex group (JEV and WNV) [15, 16]. Pre-existing immunity to JEV was also associated with an increased occurrence of symptomatic DENV infection [12]. Because non-FcγR bearing cells were used in these studies, the sum of neutralizing activity and infection-enhancement activity, and the ADE activity of these antibodies at subneutralizing levels, has not been determined. Thus, a better understanding on cross-reactive immunity induced with JE vaccination, particularly the infection-enhancement and neutralizing activities of the cross-reactive antibodies, is expected to facilitate development of effective vaccination and preventive strategies for flavivirus disease control, including DENV and JEV infection [17–19].

Potential interactions between flaviviruses have significant public health implications because areas with high JE vaccine coverage and JEV circulation are also frequently areas with a high dengue disease burden [12]. With the rapid expansion of DENV endemic areas and emergence in previously non-endemic areas, an improved understanding of the pre-existing immunity against JEV and of the potential implications for subsequent DENV exposure is necessary. In this study, we used BHK and FcγR-expressing BHK cells to determine DENV cross-reactive activities in antibodies of adults with pre-existing immunity to JEV, and those facilitated by JE vaccination.

## Methods

### Serum samples

Serum samples were obtained from 77 Japanese adults (mean age = 38.1 ± 10.7; male:female ratio = 1.8:1) who received a single dose of Vero cell-derived inactivated JE vaccine (JEBIK V®, BIKEN, Japan) at the National Center for Global Health and Medicine (NCGM), Tokyo Japan [20]. These serum samples were obtained before vaccination and 3–5 weeks after JE vaccination during the period of 2009–2011 (Additional file 1 Table S1), during which no local DENV outbreaks were reported [20]. Samples were collected at the NCGM, and all laboratory tests were performed at the National Institute of Infectious Diseases, Japan (NIID). The study protocols were approved by the NCGM and NIID Institutional Ethics Review Board (no. 473). All samples were de-identified prior to tests conducted at the NIID. All serum samples were heat-inactivated at 56 °C for 30 min prior to use. Of the 79 participants who received the JE vaccine, 50 participants had previous immunization history against JE, however the JE vaccination history for the other 29 participants was unknown. Samples from 2 participants were excluded from this study because of insufficient sample volume. Thus, samples from 77 participants were used in this study. Because the travel history of the participants was incomplete, that data were excluded from this study.

### Determination of the levels of anti-DENV IgM and IgG antibodies by ELISA

The amount of DENV-specific IgM antibody in the serum samples was determined using an IgM capture ELISA kit (Dengue Virus IgM Capture DxSelect ELISA Kits, Focus Diagnostics, CA, USA) according to the manufacturer’s instructions. Dengue IgG ELISAs (Dengue IgG Indirect ELISA, Panbio, Queensland, Australia and Dengue ELISA IgG, Vircell, Granada, Spain) were used according to manufacturer’s instructions for the detection of anti-DENV IgG antibody. The presence of DENV cross-reactive IgG antibody was defined as positive detection by at least one of these kits [21]. The index value of anti-DENV IgM and IgG ELISA was determined by dividing the average OD of each sample by the cut-off value according to manufacturers’ instruction. The cut-off value was determined by multiplying the average OD of the calibrator by the calibration factor provided by the manufacturer. Index values of <0.9, 0.9–1.1, and, >1.1 were considered negative, equivocal, and positive, respectively. Equivocals were regarded as negative.

### Determination of the levels of anti-JEV IgG antibody by ELISA

Anti-JEV IgG antibody in samples was determined by using an in-house JEV-specific IgG ELISA. A 96-well ELISA plate (NUNC, Thermo Scientific, USA) coated overnight at 4 °C with JEV antigen was used. After washing four times with 1× Dulbecco’s phosphate buffered saline, no calcium, no magnesium (1× DPBS (−)) (Life Technologies, CA, USA), and 200 μl of 0.1 % casein in 1× DPBS (−) was added into each well, and the plate was incubated at 37 °C for 2 h. After washing, 100 μl of the ten-folds diluted serum samples (1:100 to 1:25,600) were added and the plates were incubated at 37 °C for 1 h. After washing, 100 μl of horseradish peroxidase (HRP)-conjugated anti-human IgG was then added into each well, and the plate was incubated at 37 °C for 1 h. After further washing, 100 μl of tetramethylbenzidine (TMB) solution was added and reactions were terminated after 10 min with 100 μl of stop solution (1 N H_2_SO_4_). The ELISA endpoint corresponded to the highest dilution of the serum sample in which the positive to negative (P/N) ratio of the O.D. values was greater than 2. Thus, samples of P/N ratio of >2 were interpreted as positive.

### Viruses and cell lines

DENV-1 (01–44 strain), DENV-2 (TLC-30 strain), DENV-3 (NRT 09–34 strain), and DENV-4 (08–11 strain) were used in neutralizing and ADE assays against DENV [22]. JEV (Beijing-1 strain) was used in the JEV neutralizing assays. Baby hamster kidney (BHK) cells (Japan Health Science Research Resources Bank, Japan), FcγR-expressing BHK cells and Vero cells (African green monkey kidney epithelial cells) were used [21, 22]. BHK cells and Vero cells were cultured in Eagle’s Minimum Essential Medium (EMEM, Sigma), supplemented with heat-inactivated 10 % fetal bovine serum (FBS, Sigma) without antibiotics. The cells were maintained at 37 °C in 5 % CO_2_. FcγR-expressing BHK cells were cultured in EMEM, supplemented with heat-inactivated 10 % FBS and 0.5 mg/ml neomycin (G418, Roche Diagnostics GmbH Mannheim, Germany) at 37 °C in 5 % CO_2_.

### JEV and DENV neutralization assay

Neutralization tests against JEV were performed by using the 50 % focus reduction neutralization test method (FRNT_50_) as previously reported [20]. Neutralization tests against DENV were performed by the 50 % plaque reduction neutralization test (PRNT_50_) method as previously reported [22]. The presence of a NA titer was defined as the demonstration of a 50 % or greater plaque reduction at a titer of ≥1:10. The values of neutralizing antibody titers of below limit of detection (<1:10) were considered equal to the limit of detection divided by the square root of 2.

### Antibody-dependent enhancement (ADE) assay for DENV

The presence of ADE activity was determined by plaque assay. A total of 154 serum samples was diluted to 1:10 in EMEM supplemented with 10 % FBS. DENV were prepared at titers of 2000 PFU/ml for ADE assays using 10-fold-diluted serum samples. Virus-antibody mixtures were prepared by mixing 25 μl each of each DENV serotype with 25 μl of diluted serum samples. A control mixture was prepared by combining 25 μl of each DENV serotype with 25 μl of EMEM supplemented with 10 % FBS. For samples that demonstrated ADE activity at a 1:10 dilution, further tests was performed using undiluted serum samples [23]. Infection enhancement (measured as ADE activity) was tested using serum samples that had been diluted 1:10. ADE activity was defined as fold-enhancement values greater than the cut-off value (cut-off value = mean plaque count FcγR-expressing BHK cells without the addition of human serum using + 3 s.d.)/(mean plaque count of FcγR-expressing BHK cells without the addition of human serum + 3). The presence of ADE activity was defined as having fold enhancement values of >1.9 for DENV-1, DENV-2, and DENV-3 or of >1.8 for DENV-4.

### Statistical analyses

The tabulation, management, and analyses of data were performed using Microsoft Excel. Study outcomes were compared by *t*-tests, chi-squared tests, Fisher’s exact tests and Pearson’s correlation test [24, 25]. Geometric datasets were used for parametric analyses.

## Results

### Increase in positive rates of anti-DENV IgG after JE vaccination

**Fig. 1:**
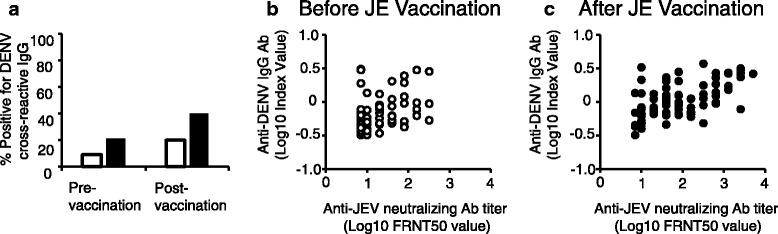
Dengue virus (DENV) cross-reactive antibodies facilitated by Japanese encephalitis virus (JEV) vaccination. **a** Percentage of vaccinees with anti-DENV IgG antibodies. Open bars indicate serum samples with no neutralizing JEV antibodies, and closed bars indicate samples with ≥ 1:10 neutralizing antibody titers to JEV. Serum samples obtained before (**b** open circles) and after (**c** closed circles) JE vaccination. An ELISA index value above 1.1 was considered positive for DENV IgG antibodies

The overall serological cross-reactivity to DENV in adult JE vaccine recipients was examined by anti-DENV IgG ELISAs. ELISA was performed using 154 serum samples obtained from participants pre- and post-JE vaccination (Additional file 1: Table S1). Prior to JE vaccination, seropositive rate of DENV IgG seropositivity was 9 % (3/34) in samples with no JE neutralizing antibodies, and was 21 % (9/43) in samples with JEV neutralizing antibodies. After JE vaccination, anti-DENV IgG antibodies was detected in 20 % (1/5) samples without JEV neutralizing activity (PRNT50 < 1:10), and in 40 % (29/72) samples with JEV neutralizing activity (Fig. 1a). Overall, the number of participants that demonstrated DENV cross-reactive IgG antibody increased from 12 to 30 after JE vaccination. DENV reactivity was defined as positive if a sample was determined to be positive by either of the two ELISA platforms used (Additional file 2: Table S2). The anti-DENV IgG index values were higher after JE vaccination (*t*-test *p* < 0.01) (Fig. 1b-c). Thus, the results indicate that the increase in DENV cross-reactive IgG antibodies after JE vaccination moderately correlated with the levels of JEV neutralizing activity.

### JE vaccination increased ADE activity to all four DENV serotypes

Vacinee serum samples at 1:10 dilutions were examined for ADE activity against each of the four DENV serotypes (DENV 1–4) by using FcγR-expressing BHK cells. The ADE activity against DENV-1 was 6 % (5/77) before vaccination and 18 % (14/77) after vaccination. For DENV2, 3 % (2/77) had ADE activity before JE vaccination and 21 % (16/77) after vaccination, for DENV3, 14 % (11/77) had ADE activity before vaccination and 31 % (24/77) after vaccination, and for DENV4, 13 % (10/77) had ADE activity before vaccination and 34 % (26/77) after vaccination. The number of serum samples observed with ADE activity to each of the four DENV serotypes significantly increased after JE vaccination (Fisher’s exact test): DENV-1 (*p* = 0.03), DENV-2 (*p* < 0.01), DENV-3 (*p* < 0.01), and DENV-4 (*p* < 0.01) (Table 1). The results indicate that cross-reactive activity to DENV increase with JE vaccination and these activities possess ADE activity to DENV.

**Table 1:** Number of serum samples with antibody-dependent enhancement (ADE) activity^a^ to each of the 4 dengue virus (DENV) serotypes, pre- and post-Japanese encephalitis (JE) vaccination

Samples	Number of serum samples that demonstrated ADE activity (Percentage of samples with ADE activity)			
	DENV-1	DENV-2	DENV-3	DENV-4
Pre-JE vaccination (*n* = 77)	5 (6 %)	2 (3 %)	11 (14 %)	10 (13 %)
Post-JE vaccination (*n* = 77)	14 (18 %)	16 (21 %)	24 (31 %)	24 (31 %)

^a^The number of serum samples observed with ADE activity to each of the four DENV serotypes significantly increased after JE vaccination (Fisher’s exact test): DENV-1 (*p* = 0.03), DENV-2 (*p* < 0.01), DENV-3 (*p* < 0.01), and DENV-4 (*p* < 0.01)

### Increase in overall ADE activity to DENV in JE vaccine recipients

The ADE activity among the four DENV serotypes and the levels of JEV neutralizing activity before and after vaccination were also examined. Serum samples were split into three groups: Group A, JE vaccine recipients that did not possess ADE activity against DENV before or after JE vaccination; Group B, JE vaccine recipients that exhibited ADE activity to DENV only after JE vaccination; and Group C, JE vaccine recipients that exhibited ADE activity to DENV both before and after JE vaccination. JEV NA titers of subjects in Group A were significantly lower before and after vaccination than those of subjects who demonstrated ADE activity to DENV after JE vaccination (Group B and Group C). The increase in JEV neutralizing activity levels in participants with ADE activity to DENV after JE vaccination was significantly higher in participants that demonstrated ADE after vaccination, in comparison to the participants that did not exhibit ADE activity to DENV (Group B = 578 ± 803, *t*-test *p* < 0.01; Group C = 980 ± 1422, *t*-test *p* < 0.01). Additionally, the number of participants that demonstrated DENV IgG cross-reactive antibodies increased in all groups (Groups A–C). Specifically, the anti-DENV IgG levels increased significantly after JE vaccination in Group B as compared to those in Group A (Group A_POST_ = 0.8 ± 0.3, Group B_POST_ = 1.5 ± 0.7; *t*-test *p* < 0.01), with Group B demonstrating 15 DENV seroconversions after JE vaccination (Table 2).

**Table 2:** Antibody-dependent enhancement (ADE) activity against the four dengue virus (DENV) serotypes in each of the samples obtained from the 77 participants, pre- and post-Japanese encephalitis (JE) vaccination and corresponding JEV neutralizing antibody (NA) levels

ADE activity in each of the serum samples to four DENV serotypes	n	JEV NA titer^a^	
		(Mean JEV NA titer ± s.d.)	
		Pre-vaccination	Post-vaccination
(A) Absence of ADE activity to DENV, both before and after JE vaccination	40	19 ± 29	153 ± 419
(B) Presence of ADE activity to DENV after JE vaccination only			
1. ADE activity to ≥3 DENV serotypes	6	98 ± 124	1130 ± 1199
2. ADE activity to two DENV serotypes	4	91 ± 153	500 ± 592
3. ADE activity to one DENV serotype	10	47 ± 64	277 ± 372
Total	20	71 ± 101^b^	578 ± 803^b^
(C) Presence of ADE activity to DENV both before and after JE vaccination			
1. ADE activity to ≥3 DENV serotypes	10	96 ± 122	1325 ± 1644
2. ADE activity to two DENV serotypes	1	40	80
3. ADE activity to one DENV serotype	4	53 ± 74	341 + 346
Total	15	81 ± 106^b^	980 + 1422^b^

^a^JEV NA titers were determined by using a 50 %-focus-reduction neutralization test

^b^The increase in JEV NA levels in participants with ADE activity to DENV after JE vaccination was significantly higher in participants that demonstrated ADE after vaccination (Group B = 578 ± 803, *t*-test *p* <0.01; Group C = 980 ± 1422, *t*-test *p* < 0.01) in comparison to those that did not demonstrate ADE activity to DENV (Group A). Samples from 2 patients were excluded due to insufficient volume for the ADE assay

Notably, the JE vaccine recipients in Group C demonstrated significantly higher anti-DENV IgG levels both pre- (*t*-test; *p* < 0.01 against Groups A and B) and post-JE vaccination (*t*-test; *p* < 0.01 against Groups A and B) than those in groups A and B. Group C also demonstrated a higher percentage of JE vaccine recipients with ADE activity against two or more DENV serotypes (73 %, 11/15), as compared to Group B (50 %, 10/20). The results, together with the observed higher JEV neutralizing activity in serum samples from Group C, suggested that pre-existing JEV immunity increased the overall immune reactivity against JEV and DENV due to facilitation by JE vaccination (Table 2).

Of the 23 participants who demonstrated anti-IgG DENV cross-reactive antibodies after JE vaccination, all had serum samples that were negative for anti-DENV IgM (data not shown), indicating that the increase in anti-DENV IgG levels after JE vaccination was not due to a recent DENV infection.

### ADE activity to DENV in undiluted serum samples after JE vaccination

The ADE activity of the serum samples with demonstrated ADE activity against DENV at 1:10 dilutions after JE vaccination were also used to determine the ADE activity in undiluted samples on FcγR-expressing BHK cells. Out of the 35 serum samples that demonstrated ADE activity to DENV at a 1:10 dilution, 26 undiluted serum samples also demonstrated ADE activity to DENV. The fold-enhancement of undiluted serum samples ranged from 2.5-fold to 14.5-fold. Of these 26 serum samples, 10 serum samples demonstrated ADE activity against a single serotype, 4 serum samples demonstrated ADE activity against two DENV serotypes, and 12 serum samples demonstrated ADE activity against three or more DENV serotypes, at undiluted levels (Table 3). Serum dilution decreases the amount of antibodies, and thus, some samples exhibited ADE activity upon serum dilution. Using undiluted serum samples, the results indicated that these serum samples possessed the potential to enhance DENV infection at biological levels.

**Table 3:** Antibody-dependent enhancement (ADE) activity against four dengue virus (DENV) serotypes in post-Japanese encephalitis (JE) vaccination serum samples that were diluted 1:10 or left undiluted

Sample no. (post-JE vaccination) (*n* = 35)^c^	Fold enhancement to DENV^a^								ADE activity to number of DENV serotypes	
	DENV-1		DENV-2		DENV-3		DENV-4			
	Undiluted	1:10-dilution	Undiluted	1:10-dilution	Undiluted	1:10-dilution	Undiluted	1:10-dilution	Undiluted	1:10-dilution
3	–^b^	–	–	–	–	–	*3.1*	*2.5*	1	1
9	–	–	–	–	–	–	2.2	*1.9*	0	1
11	*4.5* ^a^	*2.3*	*3.2*	*4.0*	*4.1* ^a^	*2.9*	2.3	*2.7*	3	4
13	*13.0*	*2.8*	*4.0*	*2.8*	*5.9*	2.1	*4.5*	*2.8*	4	3
19	–	–	–	–	*4.4*	*2.6*	–	–	1	1
20^2^	*14.5*	*2.6*	*2.8*	*3.6*	*5.3*	*2.5*	*3.3*	*2.4*	4	4
21^1^	–	–	*4.2*	*2.6*	*4.3*	*3.5*	*4.9*	*2.8*	3	3
22	*3.5*	*2.0*	–	–	–	–	–	–	1	1
31^3^	2.5	*3.0*	*6.6*	*4.8*	*2.7*	*3.2*	*4.7*	*2.4*	2	4
32^1^	–	–	–	–	2.5	*2.5*	–	–	0	1
33	*6.0*	*2.5*	*2.5*	*3.8*	*4.1*	*3.9*	*4.4*	*2.2*	4	4
43	–	–	*5.1*	*2.8*	*4.9*	*4.8*	*6.6*	1.8	3	2
47	–	–	–	–	3.2	*3.3*	1.6	*2.3*	0	2
49	–	–	–	–	*3.9*	*2.3*	–	–	1	1
51^2^	*6.7*	*2.6*	–	–	*5.0*	*2.6*	*3.3*	*2.3*	3	3
53	–	–	–	–	*3.9*	*2.0*	–	–	1	1
54	–	–	–	–	*5.0*	*3.5*	–	–	1	1
57^3^	*6.7*	*2.2*	*5.5*	*2.0*			*4.5*	*2.7*	3	3
58	*4.0*	1.9	1.5	*3.2*	*4.1*	3.2	*3.4*	*3.8*	3	3
59^3^	*4.3*	*4.2*	1.2	*2.5*	*4.0*	*3.9*	1.0	*3.4*	2	4
61	*7.3*	*2.1*	–	–	–	–	–	–	1	1
73^2^	–	–	0.4	*2.3*	*4.1*	*3.5*	1.3	*3.5*	1	3
77	–	–	–	–	*6.3*	*4.0*	*3.3*	1.8	2	1
78^1^	–	–	–	–	2.0	*2.2*	–	–	0	1
79	2.0	*2.2*	1.1	*3.6*	*5.0*	*6.7*	*3.8*	*2.7*	2	4
80	*4.0*	*2.1*	*2.5*	*2.5*	*5.4*	*3.2*	–	–	3	3
85	–	–	–	–	–	–	*6.2*	*2.6*	1	1
86^1^	1.7	*2.4*	1.0	*3.3*	2.9	*4.6*	2.3	*2.5*	0	4
89^3^	*7.0*	*4.1*	*4.2*	*2.0*	*4.8*	*5.1*	*3.3*	*3.3*	4	4
93	–	–	–	–	3.1	*3.2*	1.9	*2.4*	0	2
94^1^	–	–	–	–	–	–	*5.8*	*2.6*	1	1
95^1^	–	–	–	–	–	–	2.1	*2.1*	0	1
96^1^	–	–	*4.0*	*2.0*	*3.8*	*3.1*	*3.8*	*5.2*	3	3
100	–	–	–	–	–	–	2.4	*2.1*	0	1
110	–	–	–	–	*4.9*	*2.8*	*3.4*	*2.2*	2	2
Total number of samples	12	14	11	16	20	24	17	24		

^a^Italics indicates the presence of ADE activity

^b^–denotes an absence of ADE activity at a 1:10 sample dilution to the tested DENV serotype. Assessments of ADE activity in undiluted samples were not performed in samples that did not demonstrate ADE activity at the 1:10 dilution

^c^The numbers in superscript correspond to the number of serotypes to which a sample had ADE activity prior to JE vaccination in this study

### Absence of NA to DENV in serum samples collected from JE vaccine recipients

The DENV neutralizing activity against all four serotypes of the serum samples obtained from 77 JE vaccine recipients was determined using BHK and FcγR-expressing BHK cells. Out of the 77 JE vaccine recipients, only one (vaccine recipient #73) exhibited neutralizing activity against DENV-1 in the serum samples collected both before and after JE vaccination (BHK cells, PRNT_50_ DENV1_PRE_ = 160, PRNT_50_ DENV1_POST_ = 160; FcγR-expressing BHK cells, PRNT_50_ DENV1_PRE_ = 40, PRNT50 DENV1_POST_ = 40). Subject #73 also exhibited ADE to DENV-1, pre- and post-JE vaccination, from serum dilutions of 1:160 to 1:2560. Pre- and post-JE vaccination fold-enhancement values to DENV1 of subject #73 ranged from 2.0 to 5.9. The results suggest that participant #73 was previously infected with DENV-1. With the exception of participant #73, the absence of DENV neutralizing activity against all four DENV serotypes in our samples suggests that prior exposure to DENV was likely limited in the JE vaccine recipients of this study. Thus, although the ADE activity increased, DENV cross-reactive NAs were overall absent in this study.

### Anti-JEV IgG endpoints were higher than anti-DENV IgG endpoints in samples that were positive for anti-DENV IgG

Twelve of the serum samples collected prior to JE vaccination and positive for anti-DENV IgG antibodies were selected, and the anti-JEV IgG ELISA endpoints of these 12 serum samples were compared with their corresponding anti-DENV IgG ELISA endpoints (Additional file 3: Table S3). The JEV endpoints in all 12 samples were higher than those for DENV (mean JEV endpoint = 9600 ± 9887, mean DENV endpoint = 600 ± 618; *t*-test *p* < 0.01). The results demonstrated that in these serum samples, the levels of JEV antigen-reactive IgG were more than 10 times higher than those of DENV antigen-reactive IgG. This suggested that the presence of DENV cross-reactive antibodies in these participants was likely facilitated by JE vaccination, rather than of natural DENV infection.

## Discussion

We found that the increased occurrence of DENV cross-reactive antibodies was most pronounced in individuals with higher levels of JEV neutralizing activity. Correspondingly, the number of participants with no cross-reactive activity to DENV exhibited lower levels of JEV neutralizing activity. Other investigators have reported that cross-reactive antibodies induced by JE vaccination induced antibodies that neutralizes other flaviviruses including WNV and DENV [9, 14], while other groups have demonstrated the absence of neutralizing antibodies to other flaviviruses immunization against JE [15]. These studies however, used non-FcγR-expressing cells as assay cells. Since non-FcγR-expressing cells detects only neutralizing activity and not ADE, only cross-reactive neutralizing activity was reported [22]. Thus, the biological relevance of cross-reactive antibodies at subneutralizing levels was not determined. As antibodies induced at sub-neutralizing levels**,** efficient uptake of these virus-immune complex via the FcγR-bearing cells can increase both the number of cells infected and the virus yield [26, 27]. ADE assays using FcγR-bearing cells have proved useful for functional studies of the receptor, and in the determination of ADE activity of human sera and for defining the role of antibodies in DENV pathogenesis [28, 29]. In our study, FcγR-expressing BHK cell lines detected both neutralizing activity and ADE activity. We demonstrated that flavivirus cross-reactive antibodies were induced after immunization, and these subneutralizing cross-reactive antibodies enhanced DENV infection. Given that neutralizing antibodies have been speculated to play a dominant role in defining infection outcomes during the early phase of virus entry, they may serve to block cell attachment of virus-immune complexes to a non-FcγR-bearing cell, or inhibit virus intracellular fusion after internalization [27, 30, 31]. The FcγR may also serve a regulatory role in viral entry, and intracellular fusion and virus production, by initiating cellular responses mediated by differential Fc-effector functions, which depends on the balance of neutralizing and ADE.

Undiluted serum samples may better reflect in vivo conditions than the use of diluted samples [23]. In this study, ADE activity against all four DENV serotypes was determined by using undiluted serum samples in this study. Of note, undiluted serum samples with high JEV neutralizing activity titers also exhibited ADE activity to DENV in undiluted serum samples (Table 3). Serum dilution dilutes the amount of total antibodies, resulting in lesser amount of cross-reactive antibody. Using convalescent serum samples from DENV patients, an ADE titration curve demonstrated: (1) virus neutralization at lower dilutions, followed by (2) ADE at higher dilutions, and finally (3) absence of cross-reactivity at even higher dilutions [23]. Serum samples with subneutralizing levels of antibodies exhibited patterns of (2) and (3), in the ADE titration curve. In this study, none of the samples demonstrated cross-reactive neutralizing antibody to DENV. Thus, in some undiluted serum samples that demonstrated ADE activity, further dilution of these samples could have further led to the decrease in, or even absence of cross-reactive antibodies [patterns (2) and (3)] (Table 3). Although the association between ADE levels and severity of DENV infection needs further clarification, previous studies have suggested that ADE in undiluted samples confers higher viremia, which is a prelude to severe DENV symptoms [32–34].

In mice, JE vaccination induced DENV cross-protective immunity and virus neutralizing activity [14]. Neutralizing activity against JEV induced by a prior JE vaccination was also demonstrated to possess neutralizing activity against DENV [14]. Studies have also shown that JE vaccines have limited effect on the severity of dengue disease [35]. However, there is limited and inconclusive evidence regarding pre-existing immunity to JEV and subsequent symptomatic or severe presentation of DENV infection [12, 36]. In flavivirus infection, the ability to mount an early and vigorous antibody response is associated with better clinical outcomes, indicating that anamnestic, anti-flavivirus immune response induced by prior DENV and JEV infection, for example, is important in conferring protection, or comparatively milder disease outcomes [37–39]. In this study, history of past JE and yellow fever (YF) vaccine immunization and travel history was not available. Thus, it was not possible to conclude the contributive effects of prior vaccination or possible exposure to flavivirus during travel on the background immunity. Related discrepancies due to background immunity could be attributed to the subsequent host response to immunization and, in turn, may have contributed to the induction of ADE activity to DENV after immunization against JE in some of the participants. Although there is an association with the magnitude of ADE activity and disease severity, the mechanisms of ADE in vivo remains elusive. Other underlying biological factors include cellular immunity, viral pathogenesis, and host genetic background, all of which are speculated to be involved in the pathogenesis of severe dengue. In this study, we used FcγR-expressing BHK cells to determine the levels of ADE activity in the absence of other myeloid-specific receptors that may cooperatively facilitate infection and mediation of innate immunity. Presence of ADE antibodies may be one of the contributing factors to disease severity, however, subsequent studies using FcγR and other myeloid-specific receptors would better define the mechanisms of these antibodies in ADE-mediated immunopathology.

The JE vaccine recipients in this study may be generally characterized as having no prior DENV exposure (i.e., samples collected from a non-DENV endemic area with an absence of DENV NA). Given that JEV continues to co-circulate with DENV in endemic areas, samples in such areas may demonstrate immunity to DENV which is facilitated by either DENV or JEV infection. In this study, the effect of JE vaccination was determined using pre- and post-JE vaccination samples obtained from a non-DENV endemic area. The possibility of recent DENV infection was excluded because all vaccinees were negative for anti-DENV IgM antibodies, and the anti-JEV IgG titers were comparatively higher in these vaccinees as compared to the levels of anti-DENV IgG (Additional file 3: Table S3). Thus, the results indicate that the DENV cross-immunity in this study was induced by JE vaccination. Taken together, our results support the hypothesis that DENV cross-reactive immunity facilitated by JE vaccination may plays two competing roles: (1) at sub-neutralizing levels, these antibodies enhances DENV infection, potentially leading to symptomatic infection and severe disease presentation, and (2) JE vaccination elicits a flavivirus amnestic response that is important for disease protection, which may result in milder disease outcomes. As ADE may reflect some aspects of DENV infection in vivo, our results suggest the necessity of further studies on the long-term impact of flavivirus vaccination on DENV clinical outcomes.

## Conclusions

In summary, JE vaccination induced DENV cross-reactive antibodies, and at sub-neutralizing levels, these antibodies demonstrated ADE to DENV. Additionally, our study highlighted that cross-reactivity to DENV is associated with high levels of JEV NAs and the DENV cross-reactivity is further facilitated by JE vaccination. Our study suggests that the immunological background and interplay between JEV and DENV influences the landscape of heterologous flavivirus immunity, with JE vaccination facilitating and setting the critical threshold of ADE and neutralizing activities of DENV cross-reactive antibodies.

## Additional files

Additional file 1: Table S1. JEV NA in 77 Japanese adults pre- and post-JE vaccination. (DOC 35 kb)
Additional file 2: Table S2. Detection rates of anti-DENV IgG antibody using Panbio and Vircell kits. (DOC 28 kb)
Additional file 3: Table S3. Anti-JEV and anti-DENV IgG endpoint titers after JEV vaccination as determined by ELISA. (DOC 35 kb)

## Abbreviations

ADE: Antibody-dependent enhancement
BHK: Baby hamster kidney
DENV: Dengue virus
JEV: Japanese encephalitis virus
NA: Neutralizing antibody
WNV: West Nile virus
