# Supplementary material for: Japanese encephalitis vaccine-facilitated dengue virus infection-enhancement antibody in adults
Source: BMC Infect Dis. 2016 Oct 18;16:578. doi: 10.1186/s12879-016-1873-8 (PMC5070094; doi:10.1186/s12879-016-1873-8)
Supplement: Additional file 1: Table S1. — JEV NA in 77 Japanese adults pre- and post-JE vaccination. (DOC 35 kb) [file 12879_2016_1873_MOESM1_ESM.doc]

**Table S1** JEV NA in 77 Japanese adults pre- and post-JE vaccination

|  | Pre-JE vaccination | Post-JE vaccination |
| --- | --- | --- |
| No. of samples | 77 | 77 |
| Age, mean ± SD | 38 ± 11 | |
| Male (Number) | 50 | |
| Female (Number) | 27 | |
| JEV NA titer |  |  |
| <10 | 34 | 5 |
| 10 | 8 | 12 |
| 20 | 12 | 5 |
| 40 | 7 | 13 |
| 80 | 7 | 11 |
| 160 | 5 | 8 |
| 320 | 4 | 6 |
| 640 | −^a^ | 7 |
| 1280 | − | 4 |
| 2560 | − | 5 |
| 5120 | − | 1 |

^a^ Minus sign (−) indicates that there were no JEV NA-positive samples for the indicated titer
