# Supplementary material for: Japanese encephalitis vaccine-facilitated dengue virus infection-enhancement antibody in adults
Source: BMC Infect Dis. 2016 Oct 18;16:578. doi: 10.1186/s12879-016-1873-8 (PMC5070094; doi:10.1186/s12879-016-1873-8)
Supplement: Additional file 2: Table S2. — Detection rates of anti-DENV IgG antibody using Panbio and Vircell kits. (DOC 28 kb) [file 12879_2016_1873_MOESM2_ESM.doc]

**Table S2** Detection rates of anti-DENV IgG antibody using Panbio and Vircell kits

| JE vaccination | The number of serum samples in which anti-DENV IgG antibody was detected | |
| --- | --- | --- |
|  | Panbio | Vircell |
| Before JE vaccination | 3/77 (4%) | 12/77 (16%) |
| After JE vaccination | 7/77 (9%) | 30/77 (39%) |
