# Supplementary material for: Japanese encephalitis vaccine-facilitated dengue virus infection-enhancement antibody in adults
Source: BMC Infect Dis. 2016 Oct 18;16:578. doi: 10.1186/s12879-016-1873-8 (PMC5070094; doi:10.1186/s12879-016-1873-8)
Supplement: Additional file 3: Table S3. — Anti-JEV and anti-DENV IgG endpoint titers after JEV vaccination as determined by ELISA. (DOC 35 kb) [file 12879_2016_1873_MOESM3_ESM.doc]

**Table S3** Anti-JEV and anti-DENV IgG endpoint titers after JEV vaccination as determined by ELISA

| Vaccine recipient no. | ELISA endpoint titer | |
| --- | --- | --- |
|  | Anti-JEV IgG | Anti-DENV IgG |
| 11 | 1600^a^ | 100 |
| 20 | 6400 | 100 |
| 31 | 25600 | 1600 |
| 32 | 25600 | 1600 |
| 38 | 1600 | 400 |
| 51 | 6400 | 400 |
| 57 | 25600 | 1600 |
| 59 | 6400 | 400 |
| 73 | 1600 | 400 |
| 86 | 6400 | 100 |
| 89 | 6400 | 400 |
| 96 | 1600 | 100 |
| Mean endpoint ± s.d. | 9600 ± 9887^*^ | 600 ± 618 |

^a^ Reciprocal of the highest serum dilution that gave a specific antibody-positive reaction. P/N ratios ≥2 were regarded as positive. An asterisk indicates that the difference between the anti-JEV and anti-DENV IgG ELISA endpoint titers was significant (*t*-test *p* = 0.01)
